# Health and wellness among incoming resident physicians: A multi-domain survey

**DOI:** 10.33582/2637-4900/1003

**Published:** 2018-02-20

**Authors:** James H. Tabibian, Amanda K. Bertram, Hsin-Chieh Yeh, Joseph Cofrancesco, Nancy Codori, Lauren Block, Edgar R Miller, Padmini D Ranasinghe, Spyridon S Marinopoulos

**Affiliations:** 1 Department of Medicine, Johns Hopkins University School of Medicine, Baltimore, MD; 2 Division of Gastroenterology and Hepatology, Mayo Clinic, Rochester, MN; 3 Department of Health Policy and Management, Bloomberg School of Public Health, Baltimore, MD; 4 Department of Medicine, North Shore-LIJ Health System, New Hyde Park, NY; 5 Department of Epidemiology, Bloomberg School of Public Health, Baltimore, MD; 6 University Health Services, Johns Hopkins University, Baltimore, MD

**Keywords:** Burnout, Health behaviors, Depression, Residency, Medical students, Exercise, Alcohol, Quality of life

## Abstract

**Introduction:**

Burnout and depression are well-described in medical students and physicians and can lead to adverse personal and patient outcomes; however, their time course and risk factors remain understudied. Here, we measured multiple domains of mental and physical health and wellness and assessed gender differences among incoming physician trainees beginning residency at an academic medical center.

**Methods:**

Using a cross-sectional study design, all incoming trainees (i.e. housestaff) at Johns Hopkins Hospital received a questionnaire assessing depression, burnout, sleep, exercise, and alcohol consumption, among other domains. Standardized instruments were utilized for questionnaire development. Tests of significance were two-tailed.

**Results:**

196 of 229 incoming housestaff (86%) completed the survey, and 49% were female. A history of depression was reported in 8%, and 5.4% met criteria for at least moderate depression by Patient Health Questionnaire (PHQ-9). Females were more likely to report a history of depression than males (13% vs. 3%, p=0.02) but had similar PHQ-9 scores. Four percent of participants reported feeling they were in the wrong profession. Goal and mean sleep were 7 and 6.7 hours/night, respectively. Forty-seven percent reported exercising once/week or not at all. While mean reported weekly alcohol consumption was three drinks, participants reported consuming ≥5 drinks in one sitting on average 1.6 times in the prior 6 months, and 4% used alcohol to sleep.

**Conclusions:**

Incoming housestaff reported generally favorable mental and physical health at the beginning of residency training. However, exercise rates were low, and ill-suited alcohol consumption was noted, though infrequent. The few areas of possible improvement were largely similar between males and females. Wellness interventions might capitalize on the relatively high morale and health at the completion of medical school by helping to promote healthy habits, including regular exercise and avoidance of excess alcohol consumption, throughout future training and practice.

## Introduction

The American Medical Association Code of Medical Ethics states that physicians have a responsibility to maintain their health and wellness in order to uphold the safety and effectiveness of the medical care they provide [1]. Despite this, burnout (defined generally as a state of physical, emotional or mental exhaustion from the pressures of work and a resultant diminished interest therein), depression, and other adverse outcomes have been well-documented among medical students and practicing physicians [2–8]. These adverse physical and mental health outcomes have detrimental implications not only for the individual, but also for patients, training programs, and beyond [5,9,10,11].

While the rigors of medical training may predispose to a general decline in physical and mental health, little is known about the stage of training during which these changes might occur. In recognizing the potential consequences of these changes, wellness initiatives have been proposed as an initial means to preserve health and improve quality of life among physician trainees [12,13]. However, the development and course of burnout, depression, and other adverse physical and mental health outcomes have not been well characterized among physician trainees, particularly as they first begin residency. Therefore, the optimal timing and audience for wellness interventions are not known.

In this cross-sectional study, our aim was to assess physical and mental health and wellness among incoming interns and residents (hereinafter “housestaff”) in a wide range of specialties. In addition, we sought to identify and better understand potential gender differences which may be present.

## Methods

### Study design

After obtaining the approval of the Johns Hopkins Institutional Review Board, we conducted a cross-sectional, questionnaire-based survey of mental and physical health among house staff entering clinical residency training programs at Johns Hopkins Hospital in June 2009. Incoming house staff were invited to participate during orientation by completing a confidential, voluntary questionnaire. In addition to the questionnaire, data regarding demographic characteristics as well as non-fasting serum lipid profiles were obtained from routine University Health Services intake data. All participants provided written informed consent.

### Questionnaire

A 102-item questionnaire was used to assess eight domains of mental and physical health: depression, burnout, sleep and fatigue, exercise, self-perceived wellness, bowel habits, tobacco and alcohol consumption, and fat and fiber intake. The questionnaire was derived primarily from standardized instruments, as shown in Table 1, [14–25] and consisted of both Likert and non-Likert items. Likert items were based on a 5-point scale that ranged as follows: “not at all”, “rarely”, “sometimes”, “often”, and “very often”. A side from the PHQ-9, which, by design, refers to the prior 2 weeks, all questionnaire items were asked in reference to the prior 6 months.

To examine a comprehensive array of domains in a succinct questionnaire, the PHQ-9 and Block screeners were used in their entirety, while individual items from the other instruments were selected for inclusion in the study questionnaire. The final questionnaire took approximately 25 minutes to complete based on pilot administration among senior house staff. To ensure participant safety, PHQ-9 scores were calculated within 24 hours of data collection to allow rapid and confidential referral to mental health services for any participant meeting criteria for severe depression or suicidal ideation. Eight participants met these criteria; this represented the only instance in which study participant identification was permitted.

### Data analysis

Data were summarized as percentages for Likert items or mean (± standard deviation) for non-Likert items. Results were analyzed overall and between females and males using chi-square and t-test for categorical and continuous variables, respectively. When assumptions were not met for these statistical tests, Fisher’s exact or Wilcoxon rank-sum test were used instead as appropriate. All analyses were conducted in SAS (SAS Institute Inc., Cary, NC).

## Results

### Demographics

Of the 229 incoming housestaff, 196 (85.6%) participated in the study. Of the 196 participants, 49% were female, mean age was 30 (± 3.3) years, and 45% were married. The specialties with the largest number of participants were internal medicine (19%), pediatrics (15%), anesthesiology (13%), and surgery (10%). Additional details regarding demographic characteristics are provided in Table 2.

### Mental Health

#### Depression

Eight percent of participants reported a prior history of depression, and 6% reported currently taking antidepressant medications. Responses to the PHQ-9 revealed that 79%, 15%, 3%, and 3% of participants met criteria for no, mild, moderate, and moderately severe depression, respectively. One person with moderately severe depression indicated having suicidal ideation. With respect to gender differences, females a greater proportion of females report a prior history of depression compared to males (13% vs. 3%, p=0.02); however, there were no significant differences in the the proportion currently taking antidepressant medications (9% vs. 4%, p=0.22) or in the overall distribution of PHQ-9 scores (p=0.71). The responses to these and additional depression-related questions are presented in Table 3a.

#### Burnout

Fifteen percent of participants reported feeling “often” or “very often” that there was more work to do than they practically had the ability to do, and 16% reported feeling run down and drained of physical or emotional energy. Despite these responses, only 4% of participants reported feeling that they were in the wrong profession, and only 6% reported feeling that they were not getting what they wanted out of their job “. Notably, there were no significant differences in responses to burnout-related questions between females and males. The responses to these and additional burnout-related questions are presented in Table 3b.

### Lifestyle

#### Sleep and fatigue

On average, participants reported sleeping 6.7 hours per night and having a sleep goal amount of 7 hours per night (Table 3a). This was consistent with the majority of participants reporting both sufficient quantity and quality sleep (Table 3b). However, 4% of participants reported having used alcohol “often” or “very often” to help them sleep; conversely, 31% reported using coffee, tea, or other stimulants “often” or “very often” to help them stay awake, and 12% of participants reported almost falling asleep while driving. As with burnout, there were no significant differences in responses to sleep and fatigue-related questions between females and males. The responses to these and additional fatigue-related questions are presented in Table 3b.

#### Exercise

Exercise frequency was zero times per week for 21% of participants and only once per week for 26% of participants, though 21.1% exercised four times per week or more. Based on average weekly duration and intensity of exercise, 21% of participants were considered inactive, 34% insufficiently active, and 44% sufficiently active for substantial health benefits, according to U.S. Department of Health and Human Services guidelines, and 20% were sufficiently active for extensive health benefits[26]. Sixty six percent reported feeling that they should exercise more “often” or “very often,” and similarly, 55% felt that their physical fitness was not as good as it should be. There were no significant differences in responses to questions in these domains between females and males. The responses to these and additional exercise- and activity-related data are presented in Tables 3a and 3b.

#### Self-perceived wellness

Fifteen percent of participants reported feeling that they were not setting a good example for their patients with their own health habits “often” or “very often.” The responses to self-perceived wellness-related questions were otherwise generally favorable (Table 3b) and similar between female and male participants with one exception pertaining to the impact of medical training on sexual function; females reported feeling that their libido had been affected negatively more frequently than males (p=0.036), with 18% vs. 14% reporting this “often” or “very often”.

#### Bowel habits

Bowel habits were examined given their association with exercise, physical and mental health, and quality of life as well as the paucity of information characterizing digestive health among housestaff [27,28]. Responses to several questions suggested worse bowel habits among females, as shown in Table 3b, though the numerical differences were of uncertain clinical significance.

### Substance use and diet

#### Tobacco and alcohol

Three percent of participants reported smoking cigarettes at least once per week, while 97% were non-smokers. Mean alcohol consumption was three (±3) drinks per week. Participants reported consuming five or more drinks in a single sitting (i.e. within 2 hours) an average of 1.6 (±4) times during the past six months. Females had fewer such episodes (0.8 vs. 2.4, p=0.0040) and also generally consumed fewer alcoholic drinks per week (2 vs. 3.6, p=0.03).

#### Fat and fiber intake

Participants reported consuming 85 and 25 grams of total fat and saturated fat per day, respectively, both of which are above the upper limit of national recommendations (< 65 and 20 grams daily, respectively)[29]. Participants’ mean reported fiber intake of 19 grams per day was also below national dietary recommendations (≥25 grams daily for this demographic) [29]. There were several significant differences in dietary in take between males and females: females consumed more total fat (87 vs. 81 g, p=0.008), less saturated fat (22 vs. 27 g, p=0.001), and less fiber (16 vs. 21 g, p=0.001) per day than males (Table 3b).

### Serum lipids

Participants’ total and HDL cholesterol levels were largely unremarkable. Female participants had significantly higher serum HDL cholesterol levels (70 vs. 50 mg/dL, p< 0.0001). %: 1.99–2.06; p < 0.05), have a volume of access constantly greater than 75,000 admission per year.

## Discussion

The physical and mental health and wellness among physician trainees of has important implications not only for the individual trainee, but also for patients, training programs, and beyond, yet this has historically been a difficult subject to study. The major findings of this cross-sectional, multi-domain study of housestaff at a large, diverse academic medical center are that: 1) the health and wellness of houses taff at the beginning of their training are generally good, although a majority of study participants felt that some domains (e.g. exercise frequency, fitness level) should be better; 2) rates of mental illness, burnout, and fatigue were low among participants; 3) a notably high proportion (57%) of participants were inactive or insufficiently active by U.S. Department of Health and Human services recommendations; 4) a majority of participants consumed more total and saturated fat and less fiber than nationally recommended amounts; and 5) females and males were comparable with respect to health and wellness, with only a few differences in specific domains, including a history of, but not current, depression, self-perceived wellness, diet, and alcohol use (Table 4).

Rates of burnout, depression, and fatigue were lower in our sample than those found in other studies of house staff and medical students [3,6,9], physicians in practice [30,31] college students [32], and rates of depression were similar to the general population [31,33]. Notably, our sample also had very low rates of suicidal ideation, which has been recognized as a serious problem in the medical profession [3]. There may be several reasons for these relatively favorable findings: first, the last few months of medical school might involve lighter rotations for participants. Secondly, the start of residency may mark an exciting time associated with new opportunities for participants. Thirdly, the use of different methodologies (e.g. screening instruments) may have accounted for some differences. There may also have been factors contributing to favorable mental health scores in our sample, including a sense of camaraderie, peer and program director support, achievement of goals, contribution, and enthusiasm about being new house staff physicians [8,13]. A goal, thus, in designing wellness programs for house staff may be the preservation of the generally high morale and well being present among trainees at the start of residency.

Despite multiple indicators of well being among our sample, a few areas were potentially concerning. For example, 55% of participants felt “often” or “very often” that their physical fitness was not as good as it should be, and 66% felt they should do more exercise. This was congruent with our finding that over half of the house staff in our sample had an activity level below that recommended by the U.S. Department of Health and Human Services, and 21% did not exercise at all. Furthermore, possibly related to insufficient exercise [34,35], we found that work had a negative effect on libido significantly more often in females compared to males, although this did not appear to translate into more frequent negative impact on relationships with significant others. This finding of decreased libido, to our knowledge, has not been previously explored in physician trainees.

There may be several possibilities for the finding of insufficient exercise in our sample: lack of recreational time, inadequate emphasis on exercise and fitness at their medical school, and conservative self-rating/high personal health standards. Aside from the benefits for house staff, health care provider exercise and fitness are also associated with better patient health and medical adherence [10,11,13,36]. In this regard, it is notable that 15% of participants felt they were not setting a good example for their patients with their own health habits. Thus, taken collectively, maintenance of physical fitness and health appears to represent an area that could benefit from wellness interventions, and importantly, it was an area in which trainees reported interest and a desire to improve.

As with physician fitness, tobacco and alcohol consumption have been associated with patient health behaviors and perceptions [11,37] and were generally low in our sample. However, there were a few exceptions: 4% of participants reported using alcohol “often” or “very often” to help them fall asleep within the past 6 months. We did not explore whether this may have been due to (work-related) sleep-wake cycle disturbances or preoccupations as opposed to a pre-existing or developing pathological habit. Further research is necessary to account for this finding as well as its potential relationship to other responses such as falling asleep while driving or using coffee or other stimulants to stay awake. Although rare, excess alcohol consumption, defined as consuming 5 or more drinks at one sitting, was also present in our sample of housestaff and significantly more common in males (Table 4), raising the issue of whether drinking may have been used to help with stress management. In addition, excess fat and inadequate fiber intake compared to national recommendations was noted and was higher among female participants (for reasons which are unclear); this, together with inadequate exercise and more frequent depression, might account for their worse bowel habits compared to male participants. Similarly, in addition to the known association between HDL cholesterol and estrogen, the higher HDL among female participants may have ostensibly been related in part to their lower saturated fat intake compared to males. Thus, while both male and female incoming house staff generally exhibited favorable mental and physical health, our findings identify several areas in need of further investigation and potential intervention; moreover, follow-up (i.e. post-residency) data would be valuable to determine whether house staff perceptions of these domains may change during residency.

This study has several limitations. First, our study was cross-sectional and conducted at a single institution, which may limit generalizability. However, we achieved a response rate of 86%, and our sample encompassed a broad array of specialties and a diverse pool of incoming house staff, which we believe is likely to be representative of house staff at many other major academic medical centers. Secondly, social desirability bias (i.e. answering what house staff felt the investigators or program directors wanted to hear) might have been present; however, participants were informed that this study was both voluntary and confidential and that their data would be de-identified, thus making accurate self-reporting more likely. Thirdly, our sample size limited our ability to perform additional sub group analyses (e.g. within particular specialties) other than an analysis by gender. Lastly, the impact of internship and or residency on health and wellness cannot be assessed by this study; future studies, including longitudinal assessments, are needed and planned for this purpose.

In summary, our findings suggest that the baseline health and wellness of physician trainees beginning residency is generally good, with a few areas of concern. Differences in responses between female and male house staff were minimal. This may suggest that wellness interventions might be widely applicable across the residency class with male and female residents responding similarly to the stress of transitioning to residency. The findings of this study speak to the need to develop programs to promote existing healthy habits formed before residency and to form new healthy habits during training. Moreover, wellness interventions, including facilitating more exercise at a program level, should be timed in such a way to capitalize on the high level of morale and physical and mental health at the beginning of residency.

## Figures and Tables

**Table 1:** Standardized instruments used as a basis for the study questionnaire.

Domain	Instrument(s)
Depression	Patient Health Questionnaire-9 (PHQ-9) [14]
Burnout	Maslach Burnout Inventory [15]
Sleep and fatigue	Pittsburgh Sleep Quality index, [16] Epworth Sleepiness Scale, [17], and the Iowa Fatigue Scale [18]
Exercise	Paffenbarger Physical Activity Questionnaire [19]
Self-perceived wellness	Self-perceived health and health-related behaviors, [20] Health Perceptions Questionnaire [21]
Bowel habits	Constipation Severity Instrument [22]
Tobacco and alcohol	National Institute on Alcohol Abuse and Alcoholism, [23] Global Adult Tobacco Survey [24]
Fat and fiber intake	Block Dietary Fat and Fruit/Vegetable/Fiber screeners [25]

**Table 2:** Demographic characteristics of incoming housestaff

	Total (n=196)
**Age, years (± standard deviation)**	30 (± 3.3)
**Married**	45%
**Female**	49%
**Race**	
Non-Hispanic White	56%
Asian	29%
Black	11%
Latino	4%
Other or unknown	1%
**Department/Specialty**	
Internal Medicine	19%
Pediatrics	15%
Anesthesiology	13%
Surgery	10%
Psychiatry	6%
Radiology	6%
Emergency Medicine	5%
Obstetrics/Gynecology	5%
Neurology	5%
Ophthalmology	3%
Otorhinolaryngology	3%
Physical Medicine and Rehab	3%
Other	3%

**Table 3a:** Participant responses and results: non-Likert items.

	Female (n=97)	Male (n=99)	Total (n=196)
**Mental Health**			
**Depression**			
Mean PHQ-9 score (± SD)	3 (±4)	2.7 (±3)	2.9 (±3)
PHQ-9 score ≥10	5.50%	5.40%	5.40%
History of depression*	13%	3%	8%
Taking antidepressants	9%	4%	6%
**Lifestyle**			
**Sleep**			
Mean hours attempted per night	7.5 (± 0.9)	7 (± 0.8)	7 (± 0.9)
Mean actual hours per night	6.8 (± 1)	6.6 (± 1)	6.7 (± 1)
**Exercise and activity**			
Inactive or insufficiently active^†^	57%	53%	54%
Mean hours TV watched per week	26 (± 16)	23 (± 12)	24 (± 14)
Mean hours outdoors per week	7.6 (± 8)	8.6 (± 10)	8 (± 9)
**Substance use and diet**			
**Fat and fiber intake**			
Daily fat, grams*	89 (± 11)	82 (± 12)	85 (± 11)
Daily saturated fat, grams*	22 (± 5)	27 (± 7)	25 (± 6)
Daily fiber, grams*	17 (± 3)	21 (± 5)	19 (± 4)
Daily fruit, servings	6 (± 2)	7 (± 1)	7 (± 1)
**Tobacco and alcohol**			
Smoking (≥1 cigarette per week)	2%	3%	3%
Alcoholic drinks per week	2 (± 2)	3.6 (± 3)	3(± 3)
≥5 drinks, last 6 months*	0.8 (± 2)	2.4 (± 4)	1.6 (± 3)
**Serum lipids**			
HDL cholesterol*	70 (± 16)	50 (± 13)	60 (± 14)
Non-HDL cholesterol (mg/dL)	172 (± 28)	176 (± 32)	174 (± 30)

Based on U.S. Department of Health and Human Services guidelines.

* Significant difference between female and male participants, p<0.05.

Key: HDL, high-density lipoprotein; PHQ-9, Patient Health Questionnaire-9; SD, standard deviation

**Table 3b:** Participant responses and results: Likert items.

		Often or very oftent†		
		Female (n=97)	Male (n=99)	Total (n=196)
**Mental health**				
Burnout: Have you felt...				
	That you are in the wrong profession?	4%	3%	4%
	Misunderstood or unappreciated by co-workers?	2%	8%	5%
	That you are not getting what you want out of your job?	6%	7%	6%
	That there is more work to do than you have the ability to do?	15%	15%	15%
	that organizational politics/bureaucracy impair your ability to do a good job?	22%	8%	15%
	run down and drained of physical or emotional energy?	19%	14%	16%
**Lifestyle**				
Sleep: Have you...				
	used alcohol to help you sleep?	5%	3%	4%
	used medication to help you stay awake?	6%	2%	4%
	felt like you are making more errors as a result of fatigue?	7%	6%	6%
	felt like your quality of patient care has suffered as a result of fatigue?	5%	8%	7%
	almost fallen asleep while driving?	10%	14%	12%
	felt tired when you wake up?	22%	33%	28%
	used coffee, tea, or other stimulants to help you stay awake?	35%	28%	31%
	felt you have had sufficient sleep?	57%	59%	58%
	felt you have had good sleep quality?	61%	67%	64%
Exercise and activity: Have you...				
	felt there is no time for spiritual activity?	12%	17%	15%
	felt there is no time to spend with family?	17%	18%	18%
	felt there is no time to make healthy eating choices?	20%	19%	19%
	felt there is no time for exercise?	27%	38%	32%
	felt you should do more exercise?	66%	66%	66%
Self-perceived wellness: Have you...				
	felt you need to seek medical help or services?	2%	6%	4%
	felt your relationship with your significant other has been affected negatively?	13%	10%	12%
	felt you aren’t setting a good example for your patients with your health habits?	11%	19%	15%
	felt that your libido has been affected negatively? *	17%	13%	15%
	sought medical help when you felt you needed it? *	36%	31%	34%
	felt content with your physical health?	52%	49%	50%
	felt your physical fitness is not as good as it should be?	52%	57%	55%
Bowel habits: Have you...				
	felt abdominal pain due to a bowel movement?	2%	2%	2%
	experienced incomplete bowel movements? *	3%	1%	2%
	felt bothered by a bowel movement or by your bowel habits? *	3%	1%	2%
	had to alter your bowel habits because of your work?	9%	6%	8%

† lncludes “often” and “very often” Likert scale categories (condensed for simplicity); other Likert scale categories were “not at all”, “rarely”, and “sometimes.

* Significant difference between female and male participants when comparing across Likert scale categories (p<0.05).

Note: All questions refer to the last 6 months, on average.

**Table 4:** Summary of significant differences between males and females.

Variable	Comment
**Mental health**	
**Depression**	
History of depression	More frequent in females
**Lifestyle**	
**Bowel habits**	
Abdominal pain due to a bowel movement	More frequent in females
Incomplete bowel movements	More frequent in females
Feeling bothering by bowel movements/habits	
**Fitness and wellness**	
**Self-perceived health**	
Libido affected negatively [by medical training]	More frequent in females
Seeking medical help when needed	More frequent in females
**Food and substance**	
**Fats and fiber**	
Total daily fat intake, grams	Higher in females
Total daily saturated fat intake, grams	Lower in females
Total daily fiber intake, grams	Lower in females
**Tobacco and alcohol**	
Alcoholic drinks per week	Lower in females
Binging episodes, last 6 months	Lower in females
**Laboratory data**	
HDL cholesterol	Higher in females
